# A citizen centred urban network for weather and air quality in Australian schools

**DOI:** 10.1038/s41597-022-01205-9

**Published:** 2022-03-30

**Authors:** Giulia Ulpiani, Melissa Anne Hart, Giovanni Di Virgilio, Angela M. Maharaj, Mathew J. Lipson, Julia Potgieter

**Affiliations:** 1 Climate Change Research Centre, University of New South Wales, Sydney, Australia; 2 Australian Research Council Centre of Excellence for Climate Extremes, University of New South Wales, Sydney, Australia; 3 New South Wales Department of Planning and Environment, Sydney, Australia; 4 Australian Research Council Centre of Excellence for Climate System Science, University of New South Wales, Sydney, Australia

**Keywords:** Atmospheric science, Environmental sciences, Research data

## Abstract

High-quality, standardized urban canopy layer observations are a worldwide necessity for urban climate and air quality research and monitoring. The Schools Weather and Air Quality (SWAQ) network was developed and distributed across the Greater Sydney region with a view to establish a citizen-centred network for investigation of the intra-urban heterogeneity and inter-parameter dependency of all major urban climate and air quality metrics. The network comprises a matrix of eleven automatic weather stations, nested with a web of six automatic air quality stations, stretched across 2779 km^2^, with average spacing of 10.2 km. Six meteorological parameters and six air pollutants are recorded. The network has a focus on Sydney’s western suburbs of rapid urbanization, but also extends to many eastern coastal sites where there are gaps in existing regulatory networks. Observations and metadata are available from September 2019 and undergo routine quality control, quality assurance and publication. Metadata, original datasets and quality-controlled datasets are open-source and available for extended academic and non-academic use.

## Abstract (summary)

**Table:** 

Measurement(s)	temperature of air • relative humidity • pressure of air • atmospheric wind speed • atmospheric wind direction • rainfall rate • sulphur dioxide • carbon monoxide • nitrogen dioxide • tropospheric ozone • pm10 • pm2.5
Technology Type(s)	weather station • air quality station
Sample Characteristic - Environment	urban weather and pollution
Sample Characteristic - Location	Sydney, Australia

## Background & Summary

With cities and megacities expanding both in density and sprawl around the world, and with almost 70% of the global population expected to be urbanized by 2050^1^, it is essential to understand the meteorological and air quality dynamics occurring within the urban canopy layer in a holistic way. In urban environments, the thermal budget and the radiative forcing are dramatically altered by extensive replacement of natural surfaces with man-made, heat-storing materials, by anthropogenic emissions, and by wind-breaking and canyon effects caused by urban roughness. Their collective impact is conducive to the development of localized hot spots for heat and pollutants, known as Urban Heat Islands (UHIs) and Urban Pollution Islands (UPIs)^2,3^, whose interplay and evolution under climate change dynamics and extreme events is poorly understood and requires dedicated monitoring^4^. This is especially urgent in Australia and other regions in the world strongly impacted by global and local warming, and weather upheavals^5^. At the same time, citizens endure this modified urban environment often with little awareness of their role in amplifying, or their potential to mitigate, negative environmental effects.

The Schools Weather and Air Quality (SWAQ) urban meteorological network was conceived to be novel and useful not just in providing research quality data, but also in stimulating citizens’, in particular school students’ and teachers’, participation and inclusion. SWAQ is a citizen science project funded by the Australian Government’s Department of Industry, Science, Energy and Resources. This project is the first of its kind in the Southern Hemisphere and creates a base monitoring network of research grade sensors covering primary schools in targeted suburbs of rapid urban expansion across the Sydney metropolitan region to complement official government networks. The data are available freely online via a dedicated website (www.swaq.org.au) for school and public use, complete with real-time visualisations. Teachers and students can thus relate how changes in pollution concentrations are driven by meteorological conditions, or how the onset of events such as bushfires, heatwaves, or thunderstorms can affect air quality in their local environment.

**Fig. 1:**
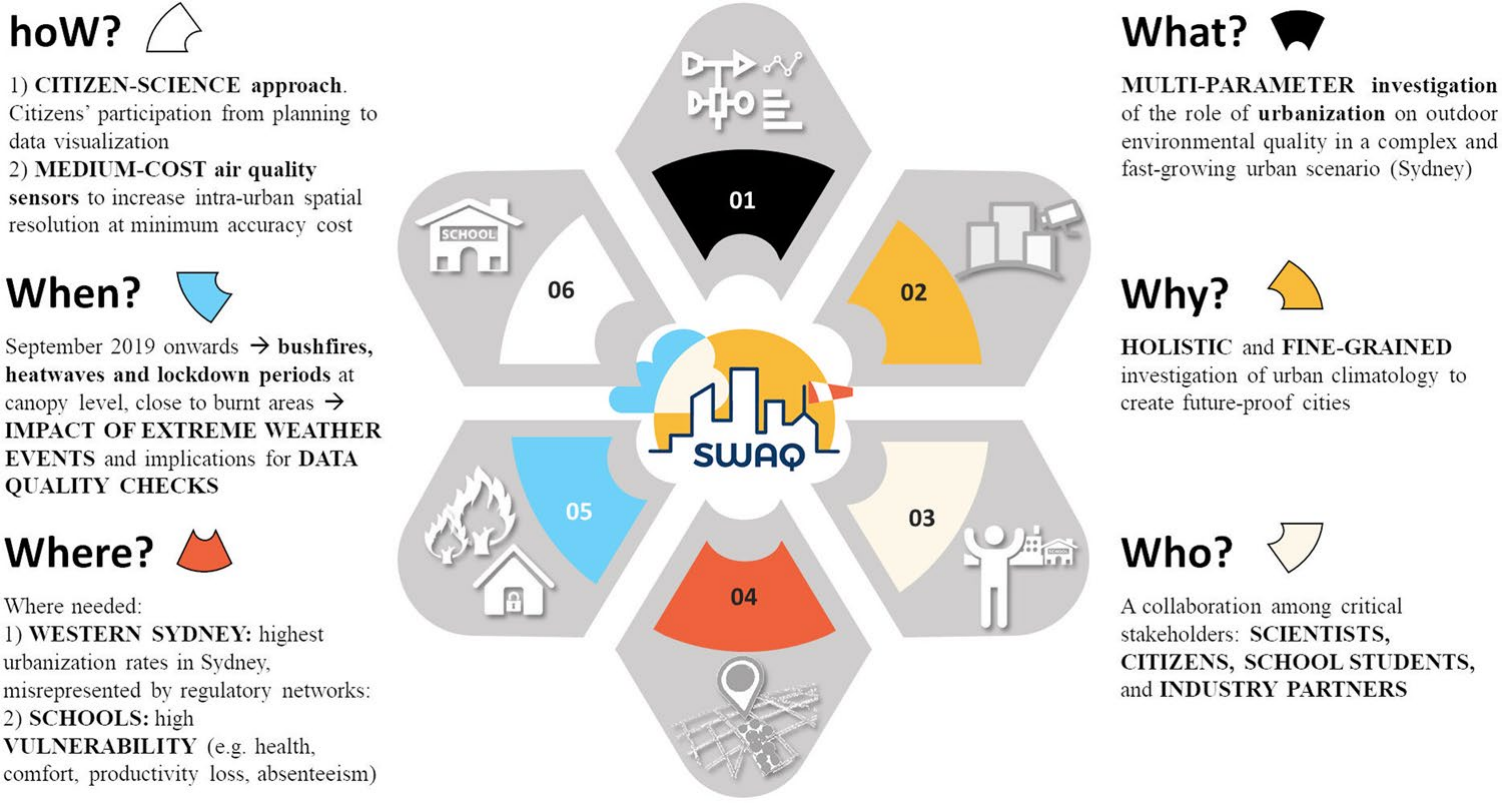
The 6 Ws of SWAQ: research questions and methodological framework.

The SWAQ dataset is of unique interest in urban science for a number of reasons that correspond to the six questions (6 Ws) that shaped its very concept, displayed in Fig. 1.

SWAQ adds to the growing number of urban meteorological networks (UMNs) deployed in the last decade worldwide, with the specific purpose of monitoring city-scale heat and air quality dynamics. Currently deployed UMNs include the Metropolitan Environmental Temperature and Rainfall Observation System (METROS) in Central Tokyo, Japan^6^, the Oklahoma City Micronet (OKCNET) in USA^7^, the Helsinki Testbed in Finland^8^, the Turku Urban Climate Research Project (TURCLIM) in Finland^9^, the Olomouc’s Metropolitan Station System in Czech Republic (MESSO)^10^, the Birmingham Urban Climate Laboratory (BULC) in the UK^11^, the HiSAN network in Tainan City, Taiwan^12^, and the MOCCA network in Ghent, Belgium^13^. SWAQ aligns with the above UMNs and devotes special attention to site documentation by following a standardized UMN metadata protocol^14^ so as to improve site representativeness, maximize comparability across UMNs, and contribute to the buildup of a consistent database.

An example of analysis performed on SWAQ data can be found in Di Virgilio *et al*.^15^ with a focus on the 2019/2020 catastrophic Black Summer wildfires^16^. SWAQ data revealed that high temperatures and low humidity, despite being classic fire weather conditions, did not have a simple direct relationship with air pollution. Instead, their impact changed depending upon the different weather systems. Intense pollution was found to move across Sydney aligned with cold fronts, as also seen with some of North America’s severe wildfires^17,18^. Further, the negative influence of wind speed on PM_2.5_ associated with dispersion and dilution was reversed at higher wind speeds owing to increased rate of advection/transport of smoke and increased wildfire activity.

Another study^19^ applied advanced statistics to demonstrate that: i) seasonal cycles are critical in shaping weather-pollution relationships, yet anthropogenic mechanisms may take over in the local presence of extensive and compact built features, ii) strong associations exist between temperature and nitrogen dioxide, relative humidity and PM_2.5_, and wind speed and carbon monoxide, iii) these interactions are not static as their nature and strength varies in time and space, modulated by the urban metabolism.

## Methods

### Monitoring equipment

Air temperature, relative humidity, barometric pressure, wind speed and direction as well as rainfall are measured at each location using Vaisala WXT536 multi-parameter weather sensors^20^. Wind is measured by a Vaisala WINDCAP® ultrasonic sensor that uses an array of three equally spaced transducers to determine horizontal wind speed and direction. Individual rain drops are detected by a Vaisala RAINCAP® piezoelectric sensor, while all other signals are recorded using capacitive sensors. The WXT536 is protected against flooding, clogging, wetting, evaporation losses and is provided with the Vaisala Bird Spike Kit to reduce the interference caused by birds on wind and rain measurements. Vaisala weather sensors are also deployed in some of the aforementioned UMNs (see “Background & Summary”), namely in the OKCNET^7^ (Vaisala WXT510 and Vaisala WINDCAP®), in the Helsinki Testbed^8^ (Vaisala WXT510), and in the BULC^11^ (Vaisala WXT520). SWAQ is based on successor models.

Six air pollutants (sulfur dioxide, nitrogen dioxide, carbon monoxide, ozone, PM_10_ and PM_2.5_) are measured at 6 locations using medium-cost, small-weight and compact-size Vaisala AQT420 air quality sensors^21^. Proprietary intelligent algorithms are incorporated to compensate for the impact of ambient conditions and aging, allowing the use of affordable electrochemical sensors in lieu of costly gas sampling and conditioning equipment for large-scale deployment. Particulate matter is measured by a laser particle counter (LPC) that quantifies the angular light scattering engendered by particles passing through the detection area, whose diameter falls in the range 0.6 to 10 μm. Particle size and concentration is estimated via digital signal processing (DSP) and is based on the spherical equivalent diameter.

Range, accuracy and resolution for each variable are detailed in Table 1, along with overall encumbrance, weight and power requirements.

**Table 1:** List of measurements and sensors’ specifications.

Symbol	Parameter	Units	Range	Accuracy	Resolution
**Meteorological data (VAISALA WXT536)**					
**Operating Voltage: 6–24 VDC, Average current consumption: 3.5 mA at 12 VDC, Protection Class: IP66, Dimensions (bird spike kit excluded): 115(w) × 238(h) × 115(d) mm, Weight: 0.7 kg**					
T	Temperature	°C	−52–60	± 0.3	0.1
RH	Relative Humidity	%	0–100	± 3 at 0–90% ± 5 at 90–100%	0.1
p	Air Pressure	hPa	600–1100	± 0.5 at 0–30 °C ± 1 at −52–0, 30–60 °C	0.1
ws	Wind speed	m/s	0–60	± 3% at 10 m/s	0.1
wd	Wind direction	°	0–360	± 3.0 at 10 m/s	1
Rain	Rainfall rate	mm/h	0–200	n.s.	0.1
**Air pollutants (VAISALA AQT420)**					
**Operating Voltage: 8-30 VDC, Average power consumption: 0.5 W, Protection Class: IP65, Dimensions: 128(w)× 185(h) × 128(d) mm, Weight: 1.25 kg**					
SO2	Sulphur dioxide (SO_2_)	ppm	0–2	± 0.05 *	n.s.
CO	Carbon monoxide (CO)	ppm	0–10	± 0.2 *	n.s.
NO2	Nitrogen dioxide (NO_2_)	ppm	0–2	± 0.025 *	n.s.
O3	Ozone (O_3_)	ppm	0–2	± 0.06 *	n.s.
PM10	Particles less than 10 µm in diameter (PM_10_)	µg/m^3^	0–5000	n.s.	0.1
PM25	Particles less than 2.5 µm in diameter (PM_2.5_)	µg/m^3^	0–2000	n.s.	0.1

*90% confidence interval in comparing with reference instrument, includes T and RH dependence in typical field conditions and sensor drift during calibration interval.

The 5 weather stations (Vaisala WXT536 sensors only, hereinafter met stations) are powered by QMP201C 12 W solar panel units, mated with 12 V lead acid or nickel-cadmium batteries^22^. QMP201C are equipped with two boxes, one for the mains power supply (Vaisala Mains Power Supply Unit BWT15SXZ) and battery regulator (Vaisala Battery Regulator QBR101) and the other for a 7 Ah backup battery. The mains power supply operates on universal AC inputs and frequencies (85 to 264 VAC and 47 to 440 Hz). The output voltage (15 VDC) is used to power the sensors as well as to charge the QBR101. The solar panel is provided with an angle-adjusting hand screw to set the site-optimized tilt precisely. Similarly, the 6 weather and air quality stations (Vaisala WXT536 and AQT420 sensors combined, hereinafter met + aqt stations) are powered by Ningbo Qixin Solar Electrical Appliance Co. SL30TU-18M (30 W peak power). The panels are connected to a 12 V lead acid or nickel-cadmium battery. All electronic ancillary components (e.g. LEDs) are regulated to maximize the autonomy time and absorb little current ( < 0.2 mA overall). One met + aqt station (STAT code “OEHS”) is powered by the mains power only as it was installed at a regulatory site where direct access to the grid was available. When neither solar nor mains power are available the battery working autonomy is approximately 4 days. Battery charging time depends strongly on solar radiation, however in good conditions it takes about 4 days to charge the battery while also powering the system.

Data transmission is performed via Multi-Observation Gateway MOG100 devices for all sensors, with unique ID and Application Programming Interface (API) key per site^23^. The MOG100 has dedicated connectors for the sensors and the solar panel, and operates as both a gateway and a logger device for Vaisala WXT530 and AQT400 Series. It comprises a GSM module for wireless communication, an additional battery regulator and input to the solar panel and a memory for data logging and local buffering. Data is stored for approximately two days, with oldest data being replaced first. The MOG100 operates at a 8–30 VDC voltage and requires an average power consumption of 80 mW. As it is enclosed in an IP66-rated weatherproof aluminum casing, it can be installed directly outdoors. This is the case for only met stations that do not require any extra battery to ensure continuing operation. For met + aqt stations, the MOG100 and additional solar power components (Vaisala Battery Regulator QBR101C and extra 12 V lead acid battery) are safely stored in a lockable IP66 weatherproof box.

Sensors and gateways are installed following calibration and testing, performed directly by Vaisala in controlled conditions and included as independent test reports. The data transfer stability (especially regarding solar energy availability), and the data quality were verified during an initial trial period that started in summer 2018. Validation was performed against the closest government station, as described in Di Virgilio *et al*.^15^. Routine maintenance visits are performed as required or otherwise at least annually. Metadata are updated accordingly. Maintenance typically includes cleaning of the radiation shields and the solar panels, battery checks and visual inspection of cable integrity, mechanical stability, and site clearness. Additional maintenance is performed on a 12–36 month interval, as detailed in Table 2, with a recalibration every two years.

**Table 2:** Sensor maintenance schedule.

Component	Activity	Typical Interval
AQT420	Visual inspection & cleaning	12–18 months
	Replace Cells & Filter	18–36 months*
WXT536	Visual inspection, cleaning & performance check	12–24 months

*Depending on the local pollution load and its impact on the Electrochemical Cells consumption/depletion rate.

Data is recorded and transmitted at 20-minute intervals by the MOG100 to the Vaisala cloud service, Beacon View^24^. The communication takes place via a 3.5 G (4-band GSM) cellular modem with integrated SIM card and ready-to-use cellular data plan through a secure HTTP protocol (HTTPS). The Beacon Cloud is a user-friendly, preconfigured, low-maintenance and scalable platform that i) ensures data integrity through embedded security features, ii) integrates and visualizes network-level data in near real-time, and iii) produces technical diagnostics on status and performance. Beacon’s open API for third-party integrations was used to establish a live link with the Climate Change Research Centre (CCRC, UNSW, Sydney) central Beacon cloud server, the CCRC high performance computing (HPC) server “Storm”, and the SWAQ website. More information is provided in the “Data Records” section.

### Siting and metadata

Optimum site allocation was determined by undertaking a multi-criterion weighted overlay analysis to explore variables that may influence data representativeness, for example, distance from major roads, and variables that may influence the need for monitoring, such as presence of vulnerable population groups, and gaps in the current regulatory monitoring networks. The Australian Bureau of Meteorology (BoM) synoptic weather station network and the New South Wales state Department of Planning, Industry and Environment (DPIE) air quality regulatory network were both assessed first to determine locations where there were currently no observation sites. Six non-sampled regions across the Sydney metropolitan area were identified. Each region was then analysed based on the following variables of interest: current and projected population density and proportion of vulnerable groups, socio-economic status including level of education and household income, density of major roads, industrial areas, and high traffic areas, areas slated for urban growth, the mode of travel to work and number of cars per household, and local climate zones (LCZ). The layers were reclassified into a common evaluation scale from 1 to 10 of suitability or environmental risk, with 10 being the most ideal location for placing a sensor. Schools in each region were then assigned a weighting between 1 and 10 and those scoring high were prioritised for the network. The risk of low outdoor environmental quality was higher in areas i) more densely inhabited, ii) largely industrial, and iii) close to sections of high traffic. Combining appropriate siting and homogeneous spatial density required careful balancing of competing requirements^25,26^. Beyond general considerations (e.g. vandalism, cost, site approvals), further challenges emerged as optimal siting is typically variable-specific^27^. Each SWAQ station measures 6 meteorological parameters through a single-body sensor and 6 of them detect 6 different air pollutants, again aggregated in a compact device, including both primary pollutants (that tend to be more localized to the emission sources) and secondary pollutants (which may accumulate further downwind). All related constraints resulted in a set of siting rules aimed at harmonizing the need for standardization and that for practical feasibility. Accordingly, all SWAQ sensors were installed:

- in homogenous urban regions, without i) sections of anomalous variation in the regional urban makeup and surrounding aspect ratio, ii) local and mesoscale climate alterations (e.g. wind tunnels or sheltered areas, cold air drainage, fog regions, transition zones or other topographically-generated climate patterns), iii) unusually wet patches in an otherwise dry area, iv) individual buildings significantly different to the average, and v) large, concentrated heat/pollution sources or sinks or local spots of altered thermo-photochemistry^14,28,29^;
- in areas falling into the WMO Class 4^27^ largely unshaded for sun elevations > 20 °C and with artificial heat sources and surfaces (e.g. buildings, asphaltic car parks, concrete walls) covering < 50% and < 30% of the surface within a circular areas of 10 and 3 m around the sensors’ screens, respectively. The selected areas were clear of high-power radio transmitters, antennas, power lines and generators that could have distorted the transmission;
- at a constant height of 2–3.5 m above ground level, on account of the dominant LCZs and thus mean Urban Canopy Layer height (z_H_). 2 m is the maximum height suggested by WMO^27^, however, adjustments of maximum + 1.5 m were adopted due to security measures and mounting requirements. This is in line with the BULC UMN in the UK^11^ where the height was fixed at 3 m.

**Fig. 2:**
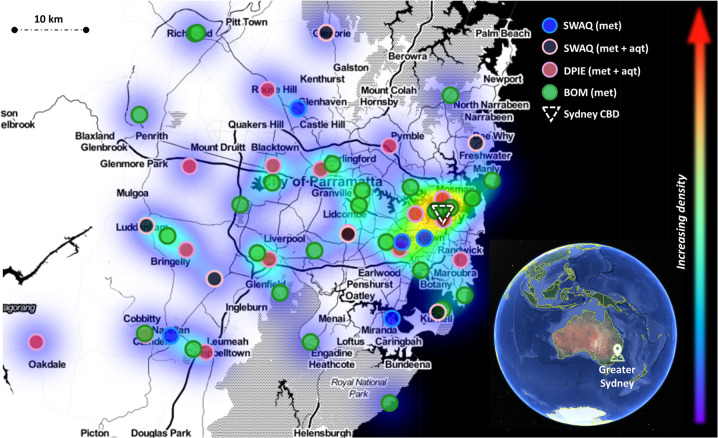
Density heatmap of meteorological and air quality observations across the Greater Sydney region. SWAQ stations (met and met + aqt) are overlapped to DPIE’s and BOM’s networks. Shades are used to visualize the density of observation sites across the region. The dashed triangle identifies Sydney’s Central Business District (CBD), while the globe in the bottom right corner shows the locations of the Greater Sydney region in the south-east corner of Australia, for reference (US Dept of State Geographer © 2021 Google Image Landsat/Copernicus Data SIO, NOAA, U.S. Navy, NGA, GEBCO).

The location of the stations is displayed in Fig. 2 with blue and black markers. Geographic and LCZ details are provided in Table 3 and, and land-use and land cover in Table 4. The minimum, average and maximum spacing are 3.7, 10.2 and 17.5 km, respectively, from −33.5995° to −34.0424° latitude and from 150.6918° to 151.2706° longitude. The SWAQ UMN complements the network of DPIE automated air quality and meteorology stations (met + aqt stations) and BoM automated weather stations (met stations) by design. These stations are aimed at evaluating synoptic-scale conditions and are thereby sited to minimize the influence of urbanization. Fig. 2 clearly shows how SWAQ UMN covers underrepresented areas by providing below-canopy observations. New sensors were installed by DPIE upon completion of our siting optimization analysis, which confirms its usefulness and representativeness in better informing the Australian health protection system.

**Table 3:** Monitoring stations: geographic coordinates, local climate zones (LCZs), and status.

	#	Public School/Site Name	STAT code	Latitude (South)	Longitude (East)	Elevation [m]	LCZ*	Date of commission/ Status
**met + aqt**	**1**	Chullora	OEHS	−33.8915	151.046	41	Large low-rise	01/09/2019/Active
	**2**	Brookvale	BROO	−33.7611	151.2706	39	Compact low-rise	01/09/2019/Active
	**3**	Glenorie	GLEN	−33.5995	151.0069	158	Sparsely built	01/09/2019/Active
	**4**	Kurnell	KURN	−34.01	151.2046	2	Compact low-rise	01/09/2019/Active
	**5**	Leppington	LEPP	−33.9593	150.8106	88	Sparsely built	01/09/2019/Active
	**6**	Luddenham	LUDD	−33.8814	150.6918	98	Open low-rise	01/09/2019/Active
**met**	**7**	Dulwich Hill	DULW	−33.9055	151.1399	33	Compact low-rise	01/10/2019/Active
	**8**	Kellyville	KELL	−33.7109	150.9579	72	Open low-rise	01/10/2019/Active
	**9**	Narellan	NARE	−34.0424	150.734	90	Open low-rise	01/10/2019/Active
	**10**	Taren Point	TARE	−34.0188	151.1231	4	Compact mid-rise	01/10/2019/Active
	**11**	Newtown	NEWT	−33.8999	151.1792	22	Compact low-rise	01/10/2019/Active

^*^Average in a 500 m radius. Data extracted from WUDAPT^43^.

**Table 4:** Land use and land cover attributes at each SWAQ site.

	#	Station	Roof height [m]*	Buildings [%]**	Road path [%]**	Other built areas [%]**	Trees [%]**	Grass [%]**	Other vegetation [%]**	Water bodies [%]**	Bare soil [%]**
**met + aqt**	**1**	OEHS	16.3	23.5 (0.1)	13.2 (7.6)	18.9 (4.7)	13.9 (24.2)	7.2 (14.8)	10.6 (17.2)	0.6 (0.0)	11.9 (9.7)
	**2**	BROO	12.8	38.2 (11.7)	10.6 (5.0)	18.0 (5.4)	20.4 (33.5)	4.7 (4.8)	6.1 (6.7)	0.1 (0.0)	1.5 (10.7)
	**3**	GLEN	7.3	7.8 (17.0)	4.7 (7.7)	5.0 (2.1)	34.4 (41.8)	27.5 (2.8)	12.9 (0.6)	0.6 (0.0)	6.6 (5.6)
	**4**	KURN	6.6	17.6 (14.0)	5.0 (11.9)	10.8 (14.1)	6.9 (15.0)	15.0 (13.5)	6.5 (8.3)	29.6 (0.0)	8.2 (1.5)
	**5**	LEPP	5.9	6.2 (7.2)	7.7 (0.2)	1.5 (3.4)	5.9 (22.6)	55.6 (13.1)	6.7 (1.5)	0.0 (0.0)	16.3 (30.5)
	**6**	LUDD	6.2	8.2 (10.9)	6.6 (6.3)	3.2 (3.7)	2.2 (0.5)	60.2 (45.4)	13.3 (7.7)	2.3 (0.0)	3.7 (2.9)
**met**	**7**	DULW	9.1	43.8 (18.3)	11.0 (17.9)	17.3 (7.5)	17.5 (20.0)	3.0 (0.7)	4.2 (3.5)	0.4 (0.0)	1.1 (4.9)
	**8**	KELL	7.1	35.6 (22.8)	15.7 (9.8)	10.5 (9.8)	10.8 (9.4)	16.8 (15.3)	5.3 (2.5)	0.0 (0.0)	4.3 (7.9)
	**9**	NARE	8.6	23.5 (35.1)	11.5 (7.9)	11.7 (8.7)	5.8 (3.1)	28.9 (14.2)	11.8 (7.8)	2.2 (0.0)	4.4 (1.5)
	**10**	TARE	9.6	27.3 (10.0)	11.3 (14.1)	19.9 (14.3)	13.7 (24.4)	12.5 (2.0)	4.0 (9.8)	4.0 (0.0)	5.5 (2.9)
	**11**	NEWT	8.9	49.5 (22.8)	4.2 (0.7)	15.9 (12.9)	23.6 (37.4)	2.9 (2.6)	2.0 (1.2)	0.0 (0.0)	1.4 (0.3)

Data extracted from Geoscape surface cover and buildings datasets^44^.

*Average in a 500 m radius.

**Average in a 500 m radius, followed by average in a 50 m radius in brackets.

## Data Records

The Beacon API was used with the “Storm” server at the University of New South Wales (UNSW) to download SWAQ raw data for analysis and archiving by running a scheduled Python script. The script converts the downloaded raw data (in XML format) as structured JavaScript Object Notation (JSON) files for permanent storage in the UNSW Data Archive. All stations’ outputs are stored as key-value pairs under the date and time stamp for each recording interval. A second script is then used to convert the json files as Comma Separated Value (CSV) files for later processing, with all stations’ outputs concatenated horizontally. The headers take the general form of “STAT_Variable”, where “STAT” is the four-character station code (see Table 3) and “Variable” indicates the measured parameter (see “Symbol” in Table 1) or the Timestamp. Data points that fail one or more quality tests (see “Technical Validation” section) are flagged. The flags are horizontally concatenated to the raw output, with a dedicated column for each station and measurement, under the heading “STAT_Variable_Flags”. All flags associated with the same data point are displayed as a semicolon-separated list.

This raw dataset, inclusive of all stations, all parameters, and corresponding flags, is stored with the identifier “YYYY-MM-DD_Raw”. Raw data is stored alongside a second csv file called “YYYY-MM-DD_Cleaned”. This is a ready-to-use dataset, quality controlled as recommended by SWAQ’s technicians. The cleaning procedure is described in the following section. Both datafiles are available from the Australian Terrestrial Ecosystem Research Network (TERN) data portal^30^. The associated Zenodo record contains the metadata files.

Date and time in both the Raw and Cleaned data files are ISO-8601-compliant.

## Technical Validation

Data quality in wireless networks like SWAQ depends on each element along the line that connects the sensed environment to the final user (e.g. power line, detectors, loggers, transmitters) and eventually determines the level of user acceptance and reliance^31^.

Quality assurance and control (QA/QC) involves different methods performed not just to ensure the quality of data, but also to preserve and prolong the service life of the equipment. QA includes periodic maintenance of stations and field sensor checks as detailed in the metadata files, whereas QC includes tests routinely performed on the data output to identify defective functioning and incorrect readings. However, some of nature’s most intriguing and life-threatening phenomena produce data that fail most automated QC tests^32^. In view of the increasing escalation of extreme weather and pollution events worldwide and especially in urbanscapes, QC procedures are designed to ensure observations of extreme episodes are not excluded.

**Fig. 3:**
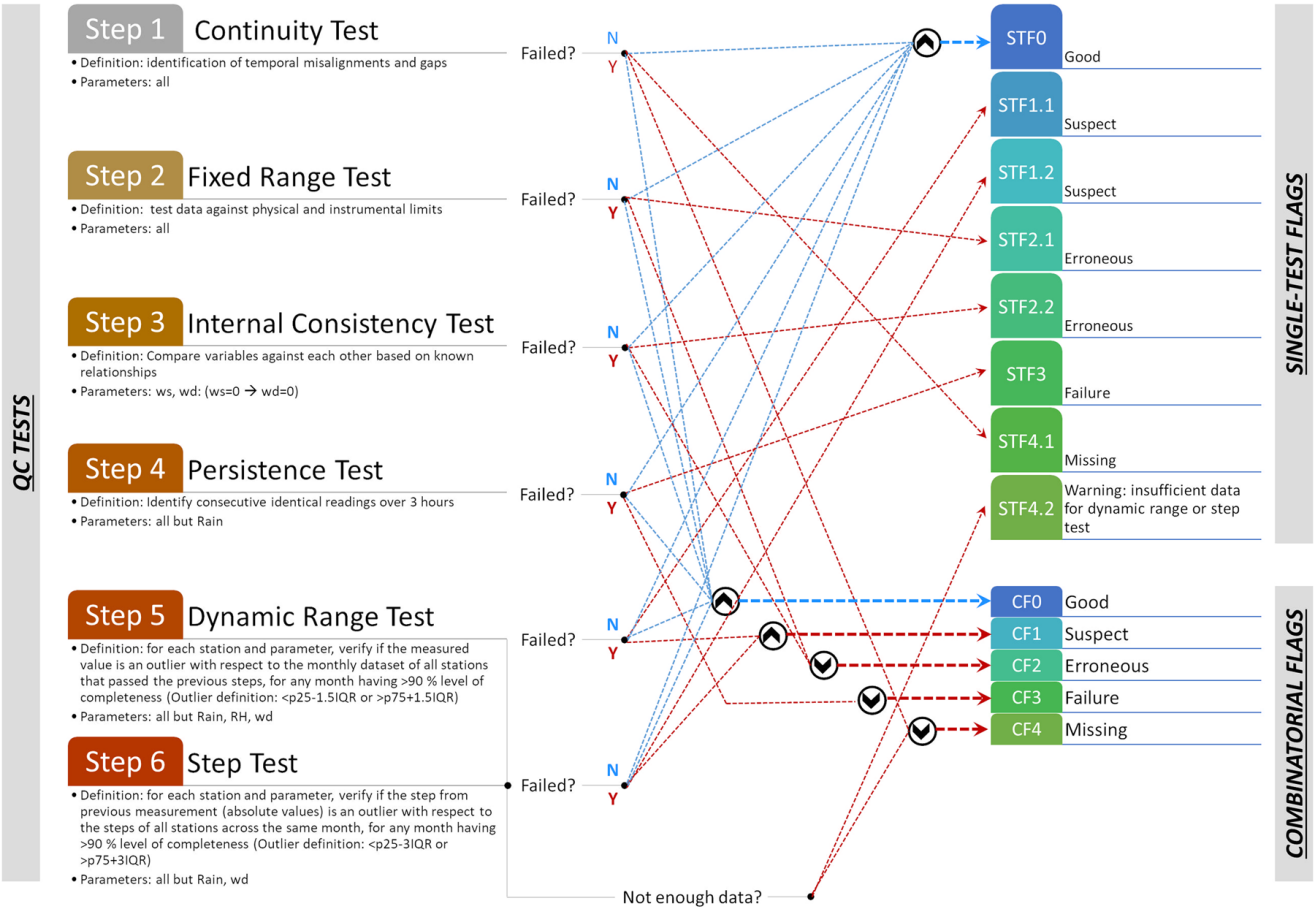
QA/QC filtering and flagging systems. Standardized icons (“ ∧ “ cap, “ ∨ “ cup) are used to represent Boolean operators (AND, OR). P25, P75 and IQR stand for 25^th^ percentile, 75^th^ percentile and interquartile range respectively.

QC on SWAQ data is performed monthly through an automated script in Python 3.9.2. In line with the Oklahoma Mesonet^33^, the Birmingham Urban Climate Laboratory network^11^, as well as the World Meteorological Organization^34^, quality control flags are used to mark erroneous and suspicious data points according to a defined set of filters. The flags supplement but do not alter the original data^35^. This entrusts the ultimate decision on deleting/preserving flagged recordings to the end user. Fig. 3 schematizes the filtering and flagging systems. In line with the 6 Ws of the SWAQ sensor network (Fig. 1), both systems are conceived to maximize data preservation and allow observation of a substantiated narrative on climatological and air quality extremes.

Filters include continuity tests, fixed range tests (on both physical and instrumental limits), dynamic range and step tests (both performed on a monthly basis), internal consistency tests (on known atmospheric relations) and persistence tests. The continuity test is used to verify that the record structure is correct, complete and without any gaps in time. The fixed range tests look for non-physical or out-of-range data. Instrumental limits were derived from equipment specification sheets, except for PM_10_. Manual inspection of PM_10_ data revealed a saturation at 3276.2 µg/m^3^, which was thus set as upper bound in fixed range tests. Dynamic range and step tests examine the relative magnitude of a given data point with respect to the statistical distribution of the same variable across the dataset. The former looks at absolute values, while the latter evaluates the rate of change of consecutive values. Lower and upper outlier thresholds for dynamic range tests and step tests are calculated monthly, rather than on annual or seasonal basis, to implicitly account for seasonal cycles and to guarantee greater comparability over times of extreme episodes, such as heat waves, droughts, thunderstorms, cold spells and bushfires. The outlier definition is stricter for step tests as compared to the standard definition applied for dynamic range tests (refer to Fig. 3), on account of Sydney’s extraordinary meteorological dynamicity, extensively reported in literature and confirmed by routine statistical analysis^36–38^. Site-specific limit bounds defined from prior experience are customary across UMNs^11,33,39^. The dynamic range test is applied to all variables but rain, RH and wd, whereas the step test is applied to all variables, but rain and wind direction. No internal consistency test is in place for rain, as the criterion entails extensive cloud cover on top of high humidity levels which would exclude most of the short-lived events that typify the region^35,40^. A 3-hour persistence criterion is applied as described in Meek and Hatfield^41^ to all variables, except rain.

The flagging system embraces a two-fold dimension, individual and combinatorial. A Single Test Flag (STF) is first applied, following the sequence in Fig. 3. The coding takes the general form of STFx.y where the first digit (x) denotes increasing severity and decreasing confidence level from good to suspicious, erroneous, and missing, whereas the second digit (y) discriminates across different filters. Months having more than 10% missing or erroneous data are issued a warning flag (STF4.2) to inform on the lack of a proper statistical sample to perform dynamic range and step tests. Removal of all STF-flagged data points does not conserve extreme events, as most localized phenomena tend to be erroneously flagged when such algorithms are taken individually^42^. The Combinatorial Flag (CF) system attempts to mitigate the risk by using Boolean operators to combine STFs. The coding takes the general form of CFx. In the CF system, only data points simultaneously failing the dynamic range test and the step test are eventually CF-flagged as suspect, since they mark sensor spikes or isolated jumps. The CF system captures the magnitude and duration of extreme events with little distortion even when all flagged recordings are removed.

The percentage of good (STF0, CF0) data is close to 90% on average, slightly lower in summer, which suggests adequate solar powering. Pollutants (especially PM_2.5_) are much more frequently flagged, given the difficulty of discerning real spikes due to local emissions or advection from erroneous measurements. However, utilizing the CF system over the STF system helps to restore episodes of consistently poor air quality. The lowest percentages are typically associated with prolonged persistence test rejection, missing values and fixed range test failure.

The original data, as stored in the “YYYY-MM-DD_Raw” datafile requires critical usage (refer to the “Usage notes” section). Conversely, the ready-to-use “YYYY-MM-DD_Cleaned” dataset is filtered in such a way to ensure both the maximum reasonable standard of accuracy and the minimum data deletion, for optimum use of the data across different urban disciplines. It involves the following sequential steps: i) replacing all negative pollutant values with zero, ii) replacing RH and wd values slightly crossing the physical boundaries with the boundaries themselves, iii) removing all data points failing the instrumental fixed range test, and iv) removing all data flagged as CFx, with x > 1.

## Usage notes

SWAQ data are cleaned according to robust QA/QC procedures and presented in a user-friendly fashion. The “YYYY-MM-DD_Raw” datafile is meant for data analysts, scientists and expert users as it maintains the raw information intact, while flagging each test failed. The “YYYY-MM-DD_Cleaned” datafile is meant for the broader public as data are already filtered based on extensive in-house expertise in urban climatology and phenomenology.

Considering all the constraints in pursuing optimal site allocation, it is highly recommended to consult metadata prior to data use. Further, it is suggested to run a final manual check aimed at identifying and removing likely unreliable data not picked up by the automatic tests, such as isolated (single site) measurements twice the average maximum across all other locations or disturbances during QA operations and recorded in the metadata or temporary sensor failures (e.g. LEPP_PM10 from 2019–10–01T00:00:00 to 2019–10–03T02:00:00).

The data and metadata files include an additional met + aqt station placed in the University of New South Wales campus (STAT code = UNSW). UNSW is part of the SWAQ network, but its siting and metadata have unique features that require special attention before use. Indeed, the station is located in a car park, under scattered trees (due to setting constraints within the University campus). UNSW data should be used and interpreted on account of local emissions of heat and pollutants, as well as potential power insufficiencies.

In addition to collecting data for urban climate and air quality research, the SWAQ network is first and foremost a citizen-centred network. The project promotes STEM in schools, by providing them with access to scientific instruments and contact with research scientists within the local context that is relevant to their community. Students learn valuable STEM skills through directly being involved in the observation and analysis of the meteorological and air quality data. School teachers and students are able to monitor conditions at their school in real time and relate how changes in local pollution concentrations are driven by variation in local meteorological conditions, or how the onset of events such as bushfires, heatwaves, or thunderstorms can affect air quality. The project has produced curriculum-aligned lesson plans that use the SWAQ data.

These lesson plans are freely available on the SWAQ website (https://www.swaq.org.au/education) and are regularly presented at science teacher’s conferences.

The data portal and visualisation of data at www.swaq.org.au/explore were developed in consultation with school students via concept testing workshops and provide timely weather and air quality data which can be freely accessed by anyone. Further, the website visualisations provide data found to be most useful and relevant to school students and members of the general public alike, with guidance on how to read the graphs and easily understandable descriptions of each of the variables presented.

## Data Availability

The code used for technical validations is publicly available in the SWAQ repository on Github: https://github.com/giuliaulpiani/SWAQ.
